# Acute Post Surgical Pain may result in chronic post surgical pain: A Systemic Review and Meta Analysis.

**DOI:** 10.12669/pjms.314.7555

**Published:** 2015

**Authors:** Wang Jiang Mei, Lin Hong Fei, Jin Hai Yan

**Affiliations:** 1 Wang Jiang Mei, Department of Anesthesiology, Children’s Hospital, Zhejiang University School of Medicine, 3333 Binsheng Road, Hangzhou 310051, China; 2 Lin Hong Fei, Department of Anesthesiology, Children’s Hospital, Zhejiang University School of Medicine, 3333 Binsheng Road, Hangzhou 310051, China; 3 Jin Hai Yan, Department of Anesthesiology, Children’s Hospital, Zhejiang University School of Medicine, 3333 Binsheng Road, Hangzhou 310051, China

**Keywords:** Acute post surgical pain, Chronic pain, Chronic post surgical pain, Risk factor

## Abstract

**Objective:**

Chronic post surgical pain (CPSP) occurres frequently (from 10% to 50%) and has serious effects on the mood and activities of patients. This study was designed to evaluate the relationship between acute post surgical pain and chronic post surgical pain.

**Methods:**

Electronic search databases included PubMed, EMBASE, Cochrane database and web of science. 9-stars Newcastle-Ottawa Scale was used to evaluate the quality of included studies. The odds ratio was used as a summary statistic index. Heterogeneity was assessed with *I*^2^.

**Results:**

We collected data from 4 case-control studies with or without chronic post surgical pain and compared those with patients who had acute post surgical pain or not. The age, sex was controlled as confounding factors. We collected 765 patients with chronic post surgical pain, of which 38.82% used to have acute post surgical pain. The risk ratio of patients with acute post surgical pain, as compared with no acute post surgical pain, was 3.10 for chronic post surgical pain (95% CI: 2.44, 3.96).

**Conclusion:**

Acute post surgical pain is a rick factor for chronic post surgical pain. We need to pay much attention to this phenomenon. However, more studies with high quality were still needed to confirm these findings.

## INTRODUCTION

Chronic post surgical pain (CPSP)is a pain of at least two months duration that develops after a surgical procedure, with exclusion of other causes.^1^ chronic post surgical pain accounts for a large rate of chronic pain, which was 33% in Norway,^2^ and occurred frequently from 10% in patients with inguinal hernia operations to 50% in patients having coronary artery bypass surgery.^3^ However, the incidence of chronic post surgical pain varies differently between operating sites, in spite of exact comparisons between studies of the same operations. So, it is important for us to explore specific approaches to prevent its occurrence.

Katz’s study, provided clear evidence of a significant predictive relationship between the intensity and quality of acute post operative pain and the development of chronic post-thoracotomy pain.^4^ In the past years, several studies have found that acute post surgical pain (acute post surgical pain) was one of the risk factors or predictors of chronic post surgical pain.^5^-^8^ However, a few studies did not find a significant association between acute post surgical pain and chronic post surgical pain 48 weeks later.^9^-^11^ Meanwhile, it was reported that blocking acute post surgical pain by analgesic approaches could minimize or prevent the chronic post surgical pain while, other research did not demonstrate that effect.^12^,^13^ So, the association between acute post surgical pain and chronic post surgical pain, is scant and still unclear.

The present systematic review aimed to summarize the current studies about the predictor ability of acute post surgical pain based on cohort study or case-control study for chronic post surgical pain for clinical works.

## METHODS

### Literature search

Electronic databases including PubMed, EMBASE, chcorane database and web of science were searched. We used the following search terms as subject headings: (“post surgical pain” OR “post-operative pain” AND acute AND chronic. All the findings were limited which were published in the last 30 years. There were no language limitations. In addition, we searched the article in the reference list of review about chronic post surgical pain to avoid missing studies.

Two reviewers evaluated the abstracts of all papers searched through search strategy mentioned above. Studies considered irrelevant by two reviewers simultaneously were excluded. A third reviewer was called in the case of controversy.

### Inclusion criteria

All studies should be about acute post surgical pain as prediction, predictor, risk factors, and correlates of chronic post surgical pain; cohort study or case-control study; studies, in which surgical patients should be followed up by 3 months at least, published between August 1990 and August 2014; the pain after surgery lasted minimum two months. The acute post surgical pain was defined as occurring in 15 days after operative.

### Exclusion criteria

Relevant studies published before 1990 were excluded; experiments based on animals; clinical evaluation of drug; studies without original data or incomplete data; case-based reports; review papers; There were no clear measurements of pain; studies of acute pain or acute pre-operative pain only.

### Data extraction and study quality

The following data were extracted for each study: the first author’s name, sample size, number of patients with chronic post surgical pain in case and control group, length of follow-up, baseline demographic information and method of diagnosis of pain. Additionally, we recorded the number of participants in each group.

The quality of studies was evaluated by the 9-stars Newcastle-Ottawa Scale (NOS).^14^ The 9-stars NOS assessed the selection, comparability, exposure of a case-control study, and outcome of a cohort study. Studies with more than 6 stars are considered as high quality ones.

### Statistical method

The Meta-analyses were conducted by Stata Version 11.0 software. The odds ratio (OR) was used as a summary statistic index and 95% confidence interval (CI) were used in the analysis. Heterogeneity was assessed with *I^2^* statistical value. An *I^2^* value more than 75% was considered as a high heterogeneity among studies. Original source for heterogeneity were evaluate by sensitivity analyses. The random-effects model would be used if the heterogeneity of studies could not be found out; otherwise, the fixed-effects model would be used. Modified Galbraith plot and Harbord weighted linear regression were used to screen for potential publication bias.

## RESULTS

### Characteristics and quality of included studies

**Fig. 1:**
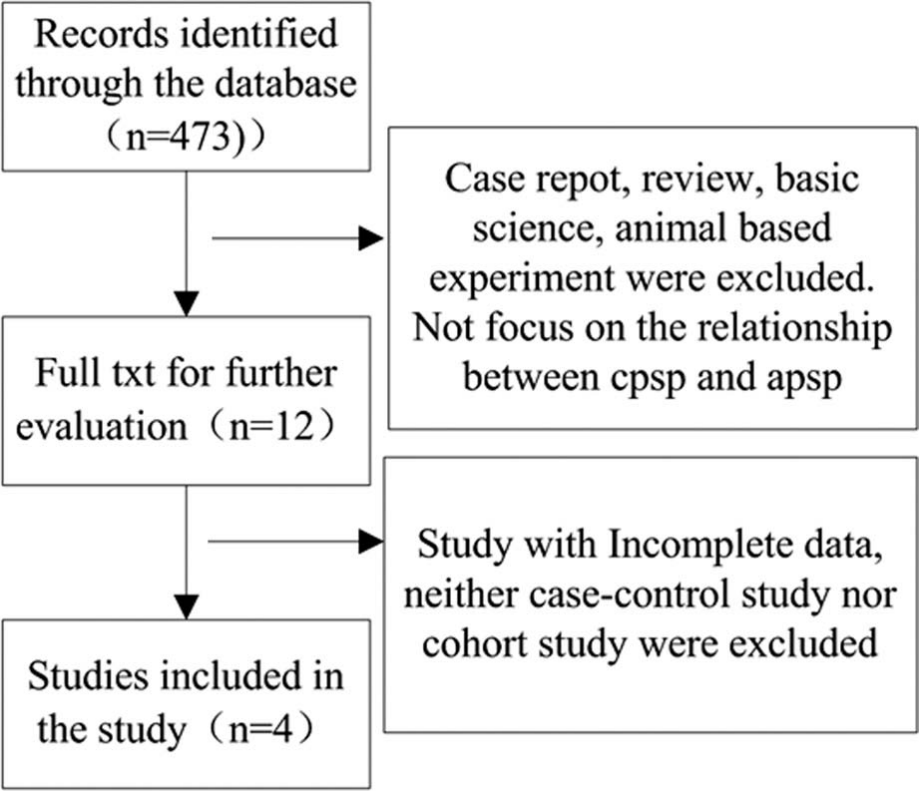
The flow of selection of studies from start to finish.

There were 4^15^-^20^ studies from 473 records included in this study. Flow chart decrypting the selection of 4 eligible studies was as follows (Fig.1).

There were 1447 participants included in this study. Among all the 4 studies, the association between acute post operative pain and chronic post surgical pain were reported. There were 4 case-control studies. Characteristics of participants in every research are outlined in Table-I. Included articles were published from 2006 to 2014. There were two studies about breast, one about hip arthroplasty and one about thoracotomy but with no clear sites. Most NOS scores of all included studies were over than 6 stars meaning the high quality.

**Table-I:** Characteristics and NOS star of participants in every study.

Author	Year	Study design	Age	Sex M/F	No. of case	Surgery category	Pain test scale	NOS star
Nikolajsen, L	2006	Case-control	18 – 90	N/A	1048	Hip arthroplasty	MPQ	6
Shadman	2014	Case-control	38.57 ± 10.66	42/113	155	Breast		
Mastoidectomy	VAS	6						
Ellen L	2006	Case-control	58.5 ± 11.7	0/95	95	Breast cancer	N/A	6
W. A. P Luijms	2006	Case-control	N/A	N/A	149	Thoracotomy	N/A	5

Insert into the blank before the section of ‘3.2 Risk of acute post surgical pain in the incidence of chronic post operative pain’.

### Risk of acute post surgical pain in the incidence of chronic post operative pain

**Fig. 2:**
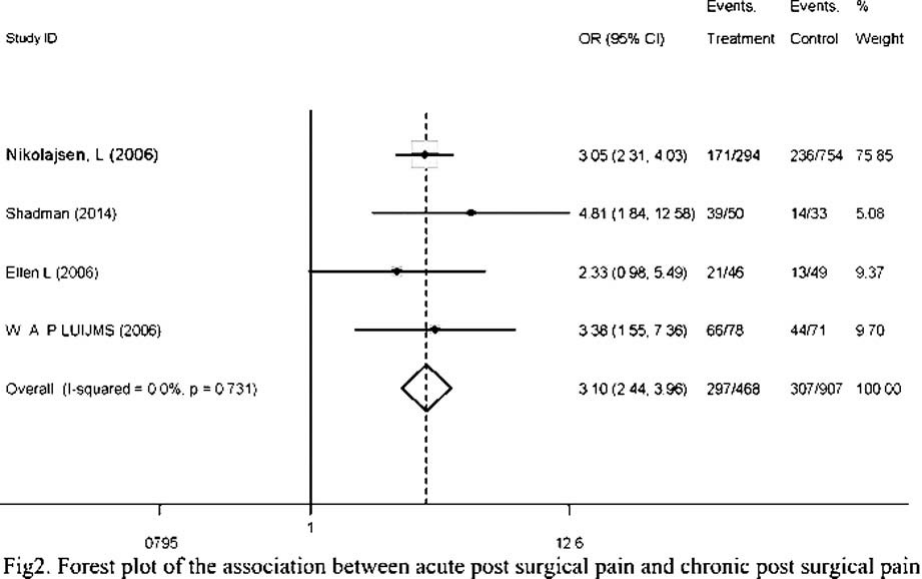
Squares are study-specific relative risk. Diamonds are summary relative risks (SRRs). Horizontal lines represent 95% confidence intervals (CIs).

Four studies reported 765 individuals suffering from chronic post operative pain in 1447 individuals. The chronic pain incidence was increased in patients undergoing surgery with acute post surgical pain, with an OR of 3.10 (95% CI: 2.44, 3.96). Meanwhile, heterogeneity test showed the value of *I* was 0% suggesting there was no heterogeneity between studies included in this study and fixed model was used (Fig.2).

### Publication bias

**Fig. 3:**
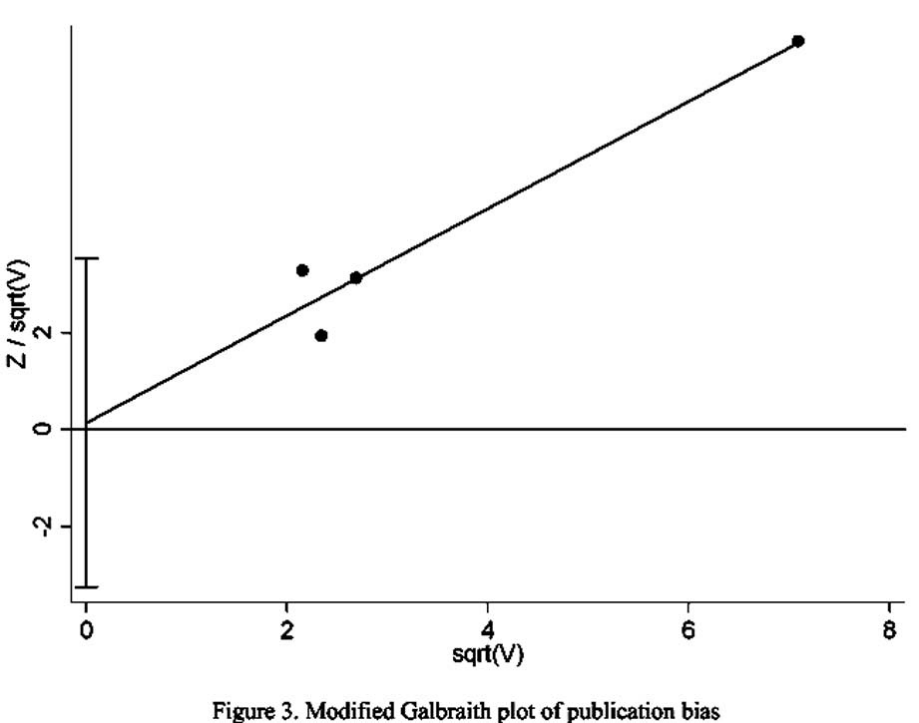
Circles are studies. Oblique line is the regression line. The 95% CI for intercept is presented as “I” form.

Harbord weighted linear regression showed no small-study effects (t=0.18, p=0.88>0.05) suggesting there was no publication bias (Table-II). Meanwhile, modified Galbraith plot declared that regression line go through starting point also meaning there was no publication bias (Fig.3).

**Table-II:** Result of Harbord weighted linear regression.

Z/sqrt(V)	Coef.	Std. Err.	t	P>t	[95% Conf. Interval]	
sqrt(V)	1.11	0.19	5.78	0.03	0.28	1.93
bias	0.14	0.79	0.18	0.88	-3.25	3.53

## DISCUSSIONS

The mechanism of chronic post surgical pain is generated by subjects of much research, most of which is conducted in animals model. So, human based evidence is lacking. It is recognized that sensitization of the central and peripheral nervous system plays an important role in the chronic post surgical pain.^21^ However, the mechanism mentioned above occurred in or around most but not all wound of patients with chronic post operative pain.^22^

Now, chronic post surgical pain has been the important clinical issue and much research has reported acute post surgical pain is the predictor of chronic post surgical pain in some prediction models. In several studies, a decrease in acute post operative pain can result in a lower incidence of chronic post surgical pain. It was reported that some interventions, reducing the dimension of hyper-sensitivity to mechanical stimulation around the wound in the abdomen, can cut down the incidence of chronic post surgical pain.^23^ Iohom^24^ reported the similar phenomenon that patients with breast surgery will have less probability of acute and then the chronic post surgical pain if they receive the treatment of continuous paravertebral block combined, acetaminophen and praecox.

Our meta-analysis of 4 included studies pointed to a result for an increased risk of chronic post surgical pain with acute post surgical pain as compared to those without acute post surgical pain. All the studies included had a high quality according to the NOS scale though there was no study was 9 stars. Heterogeneity testing indicated participants were from the same population. It is known that negative results are less likely to be published in journals, resulting in publication bias. We did not see evidence of publication bias in the Harbord weighted linear regression test, and the modified Galbraith plot.

The transformation from acute post-operative pain to chronic post-surgical pain is complex involving altered pain processing. Secondary hyperalgesia was believed to be the basis of chronic post surgical pain (CPSP). Persistent input suffered from the perioperative injury would lead to and maintain central sensitization and secondary hyperalgesia, which also would strengthen the postoperative pain and contribute to the chronic pain.^25^

### Limitations of the study.

Only four studies were included and the pain test scales were not the same. Meanwhile, the individuals in breast surgery studies were all female which may lead to the potential confounders. We also had lack of access to original source data, which would have enabled time-to-event analysis.

In conclusion, although this study has several limitations, particularly the fewer studies included in the meta-analysis, the results suggest that acute post surgical pan is associated with an increased risk of chronic post operative pain. So, more case control studies about the association between acute and chronic post surgical pain are still were needed.
